# Epidemiology and Molecular Characterization of Mycoplasmosis in Northeastern Part of Italy, 2023

**DOI:** 10.3390/pathogens15030304

**Published:** 2026-03-11

**Authors:** Caterina Signoretto, Luca Caiazzo, Gelinda De Grandi, Donato Zipeto, Paolo Gaibani

**Affiliations:** 1 Department of Diagnostic and Public Health, Microbiology Section, Verona University, 37134 Verona, Italy; 2 Department of Neurosciences, Biomedicine and Movement Sciences, Verona University, 37134 Verona, Italy; 3 Microbiology and Virology Unit, Azienda Ospedaliera Universitaria Integrata di Verona, 37134 Verona, Italy

**Keywords:** surveillance, co-infection, sequencing, typing, sexually transmitted infections

## Abstract

*Mycoplasma genitalium* (MG) is a cell wall–deficient bacterial pathogen associated with several sexually transmitted infections (STIs), including nongonococcal urethritis, cervicitis, and pelvic inflammatory disease. In the context of increasing antibiotic resistance and the challenges in clinical management, molecular epidemiological data are crucial for supporting surveillance strategies. This study aimed to assess the prevalence and genetic diversity of *M. genitalium* infections in a tertiary care hospital located in Northeastern Italy. In 2023, 2524 subjects (1622 men and 902 women) were screened using real-time multiplex PCR for the detection of major urogenital pathogens. *M. genitalium*-positive samples were molecularly characterized using a locus-typing approach based on sequence polymorphisms in the *mgpB* gene and the MG309 locus, enabling enhanced strain discrimination. Results revealed an overall positivity rate of 7.4% (118 cases), with a significantly higher prevalence in men (10.2%) than in women (2.6%), and the highest detection rate found in rectal swab specimens. Coinfections were detected in 48% of *M. genitalium*-positive subjects, most commonly involving *Ureaplasma urealyticum* (24%) and *Metamycoplasma hominis* (14%). Molecular typing on 22 *M. genitalium*-positive samples revealed significant locus-specific genetic heterogeneity, alongside the presence of a dominant cluster of 14 isolates with closely related allele profiles, suggesting the circulation of predominant local *M. genitalium* alleles within the analysed population.

## 1. Introduction

*Mycoplasma genitalium* (MG) is a sexually transmitted bacterial pathogen belonging to the class of Mollicutes and is increasingly recognised as a relevant cause of urogenital infections worldwide. It was first isolated in 1980 from the urethra of men with nongonococcal urethritis (NGU), and it is characterized by a small genome of approximately 580 kb, encoding about 525 proteins, which makes it one of the simplest self-replicating organisms known [1,2]. This minimal genetic repertoire reflects its strong dependence on the host for numerous metabolic functions and renders it a valuable model for studying essential bacterial functions. Similar to other pathogenic *mycoplasma* species, this bacterium can cause chronic infections, which can persist for up to 5 months in animal models and for more than 2 years in humans [3,4].

Unlike most bacteria, *M. genitalium* lacks a cell wall due to the absence of peptidoglycan, rendering it intrinsically resistant to beta-lactam antibiotics. Its pleomorphic structure contributes to immune evasion and enhances its survival in various host tissues. A key mechanism underlying *M. genitalium* immune evasion is the antigenic variation of surface-exposed proteins, which is mediated by diverse molecular mechanisms, including homologous and site-specific recombination [5].

*M. genitalium* is an emerging bacterium associated with sexually transmitted infections (STIs) and affects both males and females. *M. genitalium* primarily infects the urogenital tract epithelium, where it adheres tightly to host cells, leading to inflammation and tissue damage. It is a major etiological agent of several clinical conditions, including nongonococcal urethritis (NGU), non-chlamydial non-gonococcal urethritis, cervicitis, pelvic inflammatory disease (PID), and infertility [6,7].

In addition to symptomatic disease, *M. genitalium* causes asymptomatic infections, which represent a global threat to public health due to the high risk of transmission to sexual partners. *M. genitalium* transmission occurs primarily through direct genital-to-genital mucosal contact.

Symptomatic infections caused by *M. genitalium* represent a serious clinical challenge, particularly in the context of vertical transmission from the mother to the newborn, which has been associated with adverse pregnancy outcomes such as low birth weight. Coinfections appear to be associated with a greater negative impact on birth weight [8].

The pathogenicity of *M. genitalium* is mediated by several virulence factors that enable bacterial adhesion to host cells, evasion of immune responses, and induction of inflammation. Among the most relevant are the *MgPa* (P1 adhesin), accessory proteins (P140, P110), and mechanisms of antigenic variation. Recent studies suggest that *M. genitalium* may also form biofilms, further promoting persistence within the host and contributing to reduced susceptibility to antimicrobial treatments. Concurrently, the clinical management of *M. genitalium* infections is increasingly complicated by both intrinsic and acquired antimicrobial resistance. The absence of a cell wall confers intrinsic resistance to beta-lactam antibiotics, while acquired mutations have led to resistance to macrolides and fluoroquinolones, which remain the primary classes of antimicrobials used for treatment [9,10].

In recent years, the incidence of *M. genitalium* infections has increased globally, largely driven by high-risk sexual behaviours, including multiple sexual partners, sexual contact with symptomatic partners, and frequent coinfections with other sexually transmitted pathogens [11,12].

*M. genitalium* is recognized as a clinically significant STI, with an estimated global prevalence of 1–3% in the general population and substantially higher rates among high-risk groups, such as individuals with multiple sexual partners or concurrent STIs [13,14].

The epidemiology of *M. genitalium* shows marked regional variability, reflecting differences in sexual health practices, availability of diagnostic testing, and patterns of antimicrobial resistance.

To date, at least 323 different sequence types (ST) have been identified. Globally, the most predominant ones are ST-2 and ST-7, common in women and men who have sex with women, and ST-4, which is highly associated with men who have sex with men [15].

The presence of *M. genitalium* has been reported more often in patients with acute NGU than in those without it. In females, approximately 40–75% of *M. genitalium* infections are asymptomatic; however, they may develop non-gonococcal urethritis, vaginitis, cervicitis and pelvic inflammatory disease [16]. In men, clinical manifestations include acute, persistent or recurrent urethritis, dysuria, urethral discharge and proctitis [17]. In women, reported symptoms include increased or altered vaginal discharge (<50%), dysuria or micturition urgency (30%) and inter-menstrual or post coital bleeding or menorrhagia [18,19].

The most common routes of transmission are through direct genital-genital mucosal contact and penile-anal sexual contact; accordingly, a high proportion of infections are reported among men who have sex with men (MSM) [20,21].

Accurate diagnosis of *M. genitalium* infection is critical for effective for appropriate clinical management and for limiting the emergence and spread of antimicrobial resistance. Owing to its fastidious growth requirements, *M. genitalium* cannot be readily cultured, making molecular diagnostic techniques the cornerstone of laboratory identification. Diagnostic approaches for *Mycoplasma* infections include isolation of the microorganism in host cells cultures and Nucleic Acid Amplification Tests (NAATs). At present, NAATs represent the gold standard for detecting *M. genitalium*, as they amplify specific bacterial DNA or RNA sequences and offer high sensitivity and specificity [22].

Common molecular targets for NAAT-based detection include the 16S rRNA gene, the *MgPa* adhesin gene and *rpoB* (RNA polymerase subunit) gene.

Molecular typing methods are crucial for investigating the genetic variability and epidemiological dynamics of *Mycoplasma genitalium*. Given the limited number of conserved genomic regions within this organism, typing strategies have primarily focused on highly variable loci, particularly genes encoding surface-exposed proteins involved in host–pathogen interaction. Among these, the *mgpB* gene represents the most widely used target for molecular typing [15].

The *mgpB* gene encodes the major adhesin protein, a 140 kDa surface-exposed molecule that constitutes a key component of *M. genitalium*’s terminal adhesion complex, which plays a central role in bacterial attachment to host epithelial cells. The *mgpB* gene is well known to contain hypervariable regions characterized by a high frequency of single-nucleotide polymorphisms (SNPs), which generate substantial sequence diversity among strains. Consequently, *mgpB* sequence analysis has been extensively applied in epidemiological studies to assess strain diversity, monitor transmission patterns, and distinguish between persistent infection and reinfection in individual patients.

Another genetic locus commonly used for molecular typing is MG309, which encodes a putative lipoprotein located in close genomic proximity to the *mgpB* operon. Unlike *mgpB*, MG309 is characterized by variability in the number of tandemly repeated sequences, resulting in strain-specific length polymorphisms.

Currently, at least 29 distinct allelic profiles have been described, defined by variation in the number of tandem repeats composed of two trinucleotide motifs (AGT and AAT) and their specific arrangement patterns. In clinical isolates, the number of repeats units generally ranges from 7 to 17, although expansions of up to 22 repeats have been reported in specific populations [23].

This structural variability provides an additional level of discrimination that complements sequence-based approaches. The combined analysis of SNP-driven sequence variation in *mgpB* and variable number tandem repeats (VNTR) in MG309 enhances typing resolution and reduces the risk of misclassification associated with single-locus analyses. Typing schemes based on the joint evaluation of these loci have proven effective for studying the population structure of *M. genitalium*, identifying circulating strains, and elucidating sexual transmission networks [24].

In both clinical and epidemiological settings, this molecular typing approach supports contact tracing, facilitates discrimination between treatment failure and reinfection and contributes to a more accurate understanding of pathogen transmission within and between populations.

Beyond infection with *M. genitalium* alone, coinfection with other sexually transmitted pathogens plays a critical role in disease pathogenesis. Coinfections with *Ureaplasma urealyticum*, *Metamycoplasma hominis*, *Chlamydia trachomatis*, and *Neisseria gonorrhoeae* may exacerbate mucosal inflammation and promote infection persistence [25,26]. Clinically, coinfections can complicate diagnosis and negatively affect treatment efficacy, as therapeutic regimens targeting a single pathogen may be insufficient. Moreover, coinfections increase the risk of onward transmission to sexual partners, thereby facilitating the spread of multiple pathogens. Multiple concurrent infections have also been associated with increased symptom severity and higher reinfection rates, highlighting the need for integrated diagnostic and therapeutic approaches that account for simultaneous infections. Given the biological characteristics of this bacterium, extragenital screening is mandatory for its detection.

The aim of this study was to characterize the presence of *M. genitalium* among patients attending the Infectious Diseases Outpatient Clinic of a tertiary care hospital in North-East Italy. Additionally, this study sought to identify coinfections with *M. genitalium* and other sexually transmitted pathogens in order to provide a comprehensive overview of the local epidemiology and transmission dynamics of this microorganism.

## 2. Materials and Methods

### 2.1. Study Population and Sample Analysis for Sexually Transmitted Pathogens (STIs)

During 2023, a total of 5724 clinical samples were collected from 2524 subjects. The study population comprised 1622 men and 902 women. All subjects were screened for the following sexually transmitted pathogens: *M. genitalium*, *T. vaginalis*, *C. trachomatis*, *N. gonorrhoeae*, *U. parvum* and *U. urealyticum*. All participants were evaluated at the sexually transmitted diseases clinic of a tertiary care hospital located in the northeastern part of Italy. Specimens collected for STIs diagnosis included urine, rectal swabs, urethral swabs, vaginal swabs, pharyngeal swabs, cervical swabs, ocular swabs and semen. Sample and patient-related information were recorded in an anonymized database by replacing sensitive data with alphanumeric codes. No clinical data associated with these specimens were available. As this was a retrospective study, formal consent was not required.

### 2.2. Molecular Characterization of Clinical Samples for STI Pathogens

Clinical samples were screened for the detection of multiple sexually transmitted pathogens, including *Mycoplasma genitalium*, *Chlamydia trachomatis*, *Neisseria gonorrhoeae*, *Trichomonas vaginalis*, *Metamycoplasma hominis*, *Ureaplasma urealyticum* and *Ureaplasma parvum*. Pathogen detection was performed using commercial Real-time PCR “Anyplex II STI-7” (Seegene, Inc., Seoul, Republic of Korea), which simultaneously identifies of the seven major sexually transmitted pathogens.

DNA extraction was carried out using the Seegene NIMBUS automated system (Seegene, Inc., Seoul, Republic of Korea), based on magnetic beads coated with silica. Following sample lysis and digestion, the lysate was transferred to columns containing the magneti beads, to which the nucleic acids bind. The whole workflow, from nucleic acids extraction to amplification, was monitored by the addition of 20 µL of Internal Control (IC) (BIORON Diagnostics GmbH, Ludwigshafen, Germany), provided by the manufacturer, to each sample prior DNA extraction, to verify the nucleic acid extraction efficiency and to exclude PCR inhibition.

Real-Time PCR was performed using a CFX96 Real-Time Thermocycler (Bio-Rad, Hercules, CA, USA). For each reaction, 5 mL of extracted DNA was combined with 15 mL of MM (master mix) and 1 mL of internal control (IC). Negative (RNase-free water) and positive controls were included in each run.

### 2.3. mgpB and MG309 Amplification and Sequencing

Positive samples for *M. genitalium* were further analysed to characterize genetic diversity. For this purpose, a bi-locus molecular typing approach was applied, combining the study of the *mgpB* gene (SNP analysis) with analysis of the MG309 locus (short tandem repeats analysis). This two-loci strategy was selected to overcome the limited resolution of single-locus typing and to achieve higher discriminatory power.

DNA extraction from the MG-positive samples was performed using the fully automated Microlab Nimbus system (Seegene, Inc., Seoul, Republic of Korea). The presence of MG was confirmed using the Anyplex II STI-7 assay (Seegene, Inc., Seoul, Republic of Korea), according to the manufacturer’s instructions, on a CFX96 real-time thermal cycler (Bio-Rad, Hercules, CA, USA). Extracted DNA samples were stored at −80 °C until further processing.

Genomic regions of interest of *mgpB* and MG309 were amplified by conventional PCR. Primer sequences used for the PCR are summarized in Table 1.

The *mgpB* and MG309 loci were amplified in separate PCR reactions using distinct primer pairs. For the *mgpB* locus, the 281 bp region was amplified using primers *MgPa*-1 (Forward) and *MgPa*-3 (Reverse), while for MG309, an STR region of approximately 350 bp was amplified using primers 384275 (Forward) and 384655 (Reverse), as previously described [27,28].

PCR reactions were performed using 5Prime Hot Master Mix (Quantabio, Beverly, MA, USA) according to the manufacturer’s instructions, and amplifications were carried out using AB™ Applied Biosystems GENEAMP 9700 PCR System. For MG309 amplification, a touchdown PCR was performed. Obtained amplicons were verified by electrophoresis on a 2% agarose gel followed by GelRed staining. Touchdown PCR is characterized by an increased specificity, as the annealing temperature is initially set 5–10 °C higher than the standard one, and is gradually decreased over subsequent cycles. By the end of the reaction, the annealing temperature will be 2–5 °C below the usual one. For the *mgpB* gene, the thermal cycling conditions consisted of an initial incubation at 50 °C for 2 min, followed by pre-denaturation at 95 °C for 4 min, and 45 cycles of alternating incubations: denaturation at 95 °C for 30 s, annealing at 61 °C for 30 s and extension at 72 °C for 1 min. For the MG309 gene, amplification included an initial pre-denaturation step for 8 min at 95 °C, followed by 30 cycles of denaturation at 95 °C for 30 s, annealing at 45–70 °C for 45 s and extension at 72 °C for 1 min.

Amplicons were visualized by VWR™ GenoSmart 2, purified by QIAquick^®^ PCR Purification Kit (Qiagen, Venlo, The Netherlands) and sequenced by the Sanger method (BMR Genomics, Padua, Italy). Electropherogram quality was assessed using Sequence Scanner Software v2.0 software for quality assessment, while sequence analysis was performed using bioinformatics.org (for reverse complement of the reverse strand) and 4peak V1.8 software.

### 2.4. Phylogenetic Analysis of MG-Positive Samples

The resulting nucleotide sequences of *mgpB* and MG309 were analysed independently.

For each locus, sequence alignment was performed using the automated tool implemented in the PubMLST *Mycoplasma genitalium* database. For both genes, phylogenetic trees were generated separately using the maximum likelihood (ML) algorithm available within the PubMLST platform (last access 1 January 2026), applying default parameters. Branch lengths were expressed as nucleotide substitutions per site. No concatenated or multilocus phylogenetic reconstruction was performed. Instead, the two loci were evaluated as independent molecular markers to explore genetic relatedness among the analysed isolates.

## 3. Results

### 3.1. Study Population

During 2023, a total of 2524 subjects were included in this study, comprising 1622 males and 902 females. The age of the study population ranged from less than one year to 89 years. All subjects attending the sexually transmitted diseases clinic of the AOUI (University Hospital) resided in the area surrounding northeastern Italy. Out of 5724 analyses performed, the number of positive cases for at least one STI was 1604 (28%), with prevalence estimates of 996 (62.1%) in men and 608 (37.9%) in women (Table 2).

Regarding *M*

Regarding *M. genitalium*, the highest positivity rate between sites was found in the rectal swab, followed by urine and cervical swab, with 74, 25 and 8 positive samples, respectively. Overall, 118 analyses came out as positive for *M. genitalium*, revealing a positivity rate of 7.4%.

A marked predominance of cases was observed in males compared with females, with positivity rates of 10.2% (102 cases) and 2.6% (16 cases), respectively. Among the 118 samples positive for *M. genitalium*, 57 (48%) presented at least one coinfection. Coinfections involving two pathogens were observed in 35 cases (30% of total *M. genitalium*-positive samples), while coinfections involving more than two pathogens were identified in 22 cases (19%). Sex-specific differences in the distribution of coinfections were observed. Among male patients, 44 coinfected cases were identified, including 28 cases involving two pathogens and 16 cases involving more than two pathogens. Among female patients, 13 coinfected cases were detected, of which 7 involved two pathogens and 6 involved more than two pathogens.

The pathogens most frequently associated with *M. genitalium* were *Metamycoplasma hominis* and *Ureaplasma urealyticum*, detected in 14% and 24% of coinfected cases, respectively. Other sexually transmitted pathogens, including *Chlamydia trachomatis*, *Neisseria gonorrhoeae*, and *Ureaplasma parvum*, were detected at lower frequencies, each accounting for less than 10% of coinfections. No coinfections involving *M. genitalium* and *Trichomonas vaginalis* were observed.

In samples from female patients, coinfections involving *M. hominis*, *C. trachomatis*, *U. parvum*, and *U. urealyticum* were uncommon, with each pathogen accounting for less than 7% of cases.

No coinfections involving *M. genitalium* and *N. gonorrhoeae* or *T. vaginalis* were detected in female patients. Detailed data on coinfections in the overall study population, stratified by sex, are reported in Table 3.

### 3.2. Typing of M. genitalium Positive Samples

A total of 22 samples, collected from various clinical specimens including urine, rectal swabs, vaginal swabs, and cervical swabs, were analysed in this study using molecular typing analysis. All samples had been previously identified as MG-positive by diagnostic tests and were further characterized using sequence-based typing approaches.

Molecular characterization was performed by analyzing two genomic loci, *mgpB* (MG191) and MG309, which are commonly used for strain discrimination in *M. genitalium*.

### 3.3. mgpB Analysis Results

**Figure 1:**
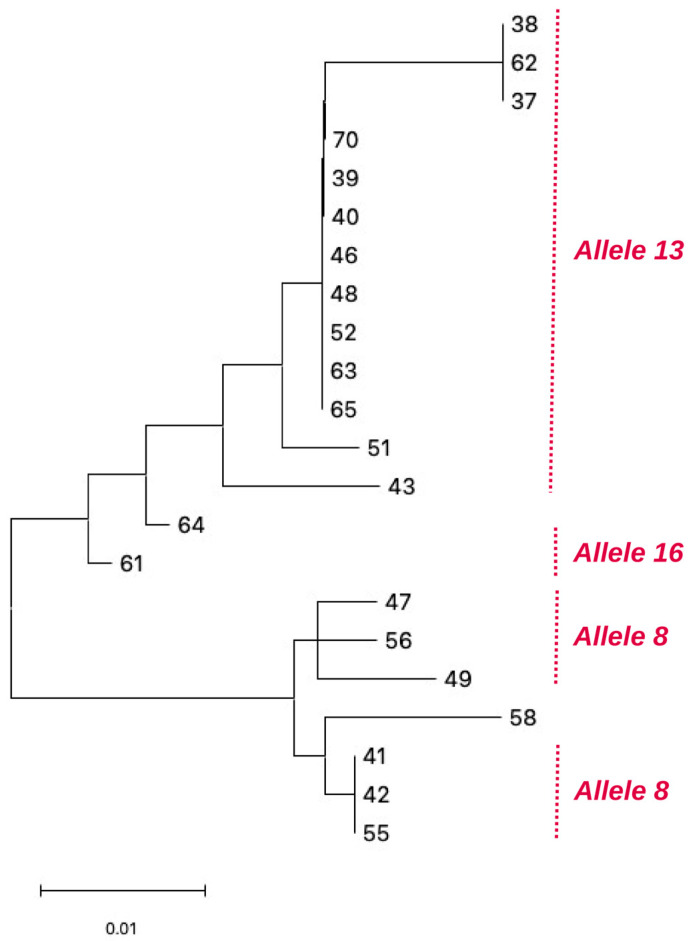
Phylogenetic tree of *Mycoplasma genitalium* isolates inferred from nucleotide sequence variation within the *mgpB* (MG191) gene. Numbers refer to the sample identification number. Scale bar represents 0.10 substitutions per site.

Allele-based phylogenetic analysis of the *mgpB* (MG191) gene was conducted to investigate genetic relatedness among the 22 *Mycoplasma genitalium*–positive samples, based on nucleotide sequence variation at this locus. Four distinct *mgpB* alleles (2, 8, 13 and 16) were identified among the isolates, indicating genetic heterogeneity within the analysed isolates. Of the 22 isolates, allele 13 was the most prevalent, detected in 12 samples (54.5%), followed by allele 8 in 7 samples (31.8%). Allele 16 was identified only in 2 samples (9.1%), while allele 2 was detected in a single isolate (4.5%). The allele distribution was reflected in the phylogenetic tree generated from the *mgpB* sequences (Figure 1), which segregated the 22 isolates into four main allele-defined lineages.

The phylogenetic tree presents two major branches: one dominated by allele 13, representing the largest cluster and comprising the majority of isolates, and a second branch containing alleles 8, 16 and 2. Short branch lengths within the major cluster indicated a high degree of sequence similarity among those isolates, whereas greater genetic distances were observed between the different allele groups.

### 3.4. MG309 Analysis Results

**Figure 2:**
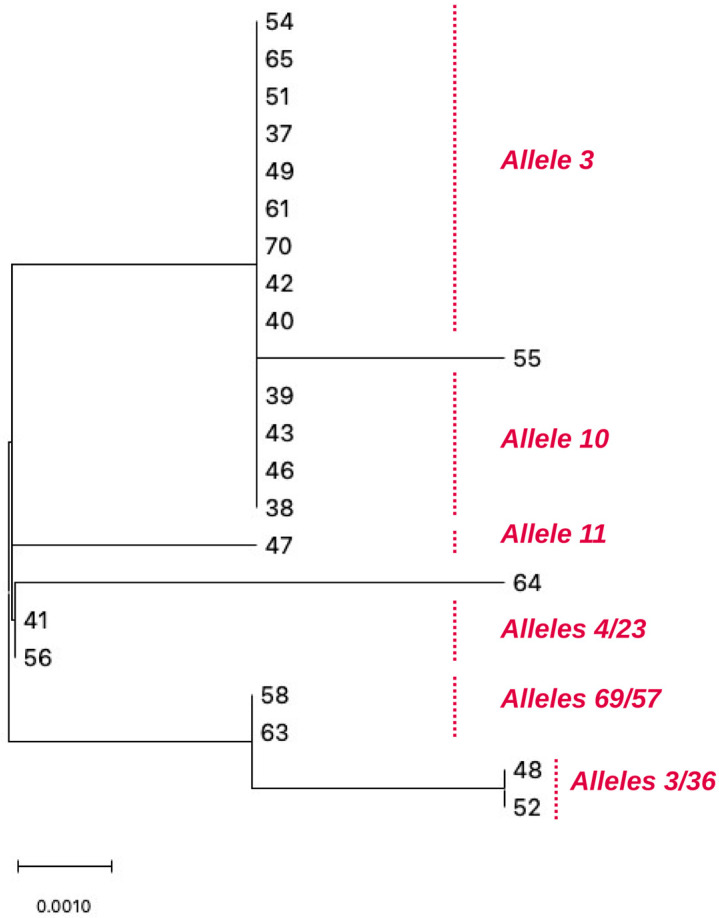
Phylogenetic clustering of *Mycoplasma genitalium* isolates based on sequence-based allele variation at the MG309 locus. Numbers refer to the sample identification. Scale bar represents 0.0010 substitutions per site.

Genetic variability within the MG309 locus was investigated using sequence-based allele typing. Sanger-generated MG309 sequences were assigned to PubMLST reference alleles, and the resulting allele profiles were used for phylogenetic reconstruction. Among the 22 analysed isolates, a total of 13 distinct MG309 alleles were identified. The most frequent allele was allele 3, detected in 6 isolates (27.3%), followed by allele 10, found in 4 isolates (18.2%) and allele 22, identified in 4 isolates (18.2%). The remaining alleles (4, 8, 11, 23, 36, 57, 68, 69) were each identified in single isolates (4.54%). The distribution of MG309 alleles and their relationships are shown in Figure 2.

The phylogenetic tree generated from MG309 nucleotide sequences revealed multiple distinct branches, reflecting the high level of allelic diversity observed. A large central cluster grouped the majority of isolates (54, 65, 51, 37, 49, 61, 70, 42, 40, 39, 43, 46 and 38), corresponding to the most frequent MG309 allele. Additional smaller clusters and isolated lineages were observed, including a paired cluster (41 and 56), a second paired cluster (48 and 52), and a closely related pair (58 and 63). Two isolates (55 and 64) appeared on long, independent branches, indicating substantial sequence divergence from the rest of the other isolates.

## 4. Discussion

The molecular epidemiological analysis of *Mycoplasma genitalium* in Northeastern Italy reveals a complex landscape of genetic diversity and transmission dynamics. The observed positivity rate of 7.4%, with a marked predominance in rectal swabs (74 cases) and among male patients (10.2%), indicates sustained transmission within defined sexual networks, particularly among men who have sex with men (MSM). These findings are consistent with clinic-based studies conducted in Sweden, where *M. genitalium* prevalence among MSM reached 10.5%, with rectal infections exceeding urethral infections, underscoring the importance of extragenital screening in high-risk populations [29]. The high burden of rectal *M. genitalium* infections reinforces current European recommendations advocating routine extragenital testing as part of comprehensive STI surveillance strategies, beyond traditional urogenital testing [30].

Nearly half of the MG-positive samples (48%) were associated with at least one additional sexually transmitted pathogen, indicating that *M. genitalium* infections frequently occur in a polymicrobial context rather than as isolated infections. This observation is epidemiologically relevant, as coinfections may facilitate transmission, complicate syndromic management, and contribute to delayed or inappropriate treatment.

Similar observations have been reported in Denmark, where high *M. genitalium* prevalence in clinical populations was often accompanied by coinfections with *Chlamydia trachomatis* or *Neisseria gonorrhoeae* and was associated with macrolide resistance [31]. These findings align with ECDC (European Centre for Disease Prevention and Control) concerns regarding overlapping STI epidemics and their role in driving antimicrobial resistance through repeated or empirical antimicrobial exposure.

From a molecular perspective, the application of a bi-locus molecular typing approach combining *mgpB* and MG309 sequence-based allele analysis enabled the evaluation of two independent genetic markers. Analysis of the *mgpB* locus revealed a limited number of allelic variants, with a dominant allele (allele 13) detected in more than half of the isolates. This limited allelic diversity resulted in the formation of a large central phylogenetic cluster, suggesting the widespread circulation of a single lineage within the study population. Such clustering is compatible with ongoing transmission within relatively closed sexual networks rather than sporadic importation events. However, the short branch lengths within this cluster indicate a high degree of genetic relatedness, thereby limiting the discriminatory power of *mgpB* when used as a standalone typing marker. In contrast, analysis of the MG309 locus revealed a higher level of polymorphism, with 13 distinct alleles identified among the 22 isolates. The corresponding phylogenetic tree showed multiple distinct branches, demonstrating that isolates grouped together based on *mgpB* typing could be further differentiated at the MG309 locus. This higher resolution is particularly relevant for public health investigations, as it enables finer discrimination of transmission chains and supports outbreak detection. Taken together, these findings support the use of a bi-locus molecular typing strategy for *M. genitalium*. While *mgpB* analysis is effective for identifying dominant circulating lineages, MG309 provides the additional resolution required to discriminate closely related strains. This combined approach is therefore well suited for epidemiological investigations aimed at distinguishing persistent infection from reinfection and for identifying potential transmission clusters. From a clinical standpoint, the observed genetic heterogeneity indicates the circulation of multiple *M. genitalium* variants rather than a single outbreak strain. This genetic diversity suggests endemic transmission rather than point-source outbreaks, reinforcing the need for sustained surveillance rather than reactive control measures. Given the absence of a cell wall in *M. genitalium* and the increasing prevalence of resistance to macrolides and fluoroquinolones, the ability to discriminate between genetically related but distinct isolates may aid in differentiating persistent infection from reinfection in both clinical practice and surveillance settings. Future studies should integrate molecular typing data with antimicrobial resistance (AMR) profiles to assess whether specific sequence types are preferentially associated with multidrug resistance.

The molecular findings of this study are further contextualized when compared with international data from other countries.

Previous investigations highlighted *mgpB* locus variability, with genetic variants frequently appearing in distinct local clusters.

Analysis of a Spanish cohort, single-locus *mgpB* typing revealed a limited number of dominant sequence types accounting for a large proportion of circulating strains, suggesting the expansion of successful local lineages [32]. Similarly, studies conducted in other European populations have reported uneven distribution of *mgpB* sequence types, with a small number of alleles predominating within defined sexual networks [33,34].

As for MG309 locus, its variability in number and arrangement of tandem repeats number has been widely recognized as a primary driver of high-resolution strain-typing; enabling differentiation of strains which would appear identical is analyzed only at the *mgpB* locus [34].

In line with these observations, this study demonstrates a limited number of *mgpB* alleles with one predominant variant, alongside with a substantially higher allelic diversity at the MG309 locus.

Some limitations of this study should be considered when interpreting the results. First, the retrospective study design restricted data collection to information available in laboratory records. Consequently, no clinical or behavioral data (such as age, symptoms, treatment history, genital tract infection history, sexual behavior, or therapeutic outcomes) were available, precluding analysis of associations between molecular profiles and clinical presentation or treatment response. Second, the study was conducted at a single tertiary care center in Northeastern Italy; therefore, the observed prevalence, coinfection patterns, and allele distributions likely reflect local epidemiological dynamics and may not be generalizable to other geographic regions or populations. Third, only a limited subset of *M. genitalium*-positive samples (22 of 118) underwent molecular typing, which may have reduced the ability to detect rare genotypes and influenced the apparent predominance of specific alleles, particularly at the *mgpB* locus. Finally, molecular characterization was restricted to two loci (*mgpB* and MG309), and although this bi-locus approach provides adequate resolution for epidemiological purposes, it does not capture genome-wide variability. Moreover, antimicrobial resistance was not directly investigated, as it was not possible to perform either genotypic or phenotypic resistance testing. As a result, no conclusions can be readily drawn regarding associations between specific genetic profiles and resistance patterns, representing an important gap given the growing clinical relevance of multidrug-resistant *M. genitalium*.

In conclusion, this study provides new molecular epidemiological data on *Mycoplasma genitalium* circulating in Northeastern Italy, highlighting both a substantial burden of infection and marked genetic heterogeneity among clinical isolates. The combined analysis of *mgpB* and MG309 loci proved to be an effective strategy for discriminating closely related strains and for characterizing local transmission patterns. The frequent detection of coinfections further supports the role of *M. genitalium* within a polymicrobial STI context. Overall, these findings reinforce the importance of integrating molecular typing and extragenital testing into routine STI surveillance to enhance understanding of *M. genitalium* epidemiology and to improve early detection, monitoring transmission networks, and mitigating antimicrobial resistance. Future studies incorporating antimicrobial resistance testing and longitudinal clinical data are warranted to overcome current limitations and to provide a more comprehensive understanding of the epidemiology and clinical impact of this emerging pathogen.

## Figures and Tables

**Table 1:** Primer sequences used to amplify *mgpB* and MG309 loci.

Region	Primer Name	F/R ^1^	Primer Sequence 5′-3′	Amplicon Size (bp)	Reference
*mgpB*	*MgPa*-1	F	5′-AGTTGATGAAACCTTAACCCCTTGG-3′	281	[24]
	*MgPa*-3	R	5′-CCGTTGAGGGGTTTTCCATTTTTGC-3′		
MG309	384275	F	5′-GTGCTAGAGAAGTGTTTCTAGGATC-3′	350	[27]
	384655	R	5′-AACTAGCAGAACGTAACCAACC-3′		

^1^ Abbreviations: F, Forward; R, Reverse.

**Table 2:** Summary of different matrices used and relative positivity rates. Percentages of positivity rates were calculated singularly for each matrix and are reported between parentheses.

Matrix	Total Analysis	Positive Rate	No. of Positive Samples to MG
Urine	2112	464 (22.0%)	25 (5.4%)
Rectal swab	1188	460 (38.7%)	74 (16.1%)
Vaginal swab	423	205 (48.5%)	5 (2.4%)
Pharyngeal swab	1173	159 (13.6%)	3 (1.9%)
Urethral swab	117	32 (27.4%)	3 (9.4%)
Cervical swab	645	276 (42.8%)	8 (2.9%)
Semen	48	6 (12.5%)	0 (0.0%)
Ocular swab	3	2 (66.7%)	0 (0.0%)
Other	15	0 (0.0%)	0 (0.0%)

**Table 3:** Distribution of *Mycoplasma genitalium*-positive samples in the overall study population (upper section) and stratified by sex (lower section). The table reports the total number and percentage of *M. genitalium*-positive cases, the proportion of coinfected samples, and the distribution of specific coinfecting pathogens. Coinf. 2 indicates *M. genitalium* coinfections involving only one additional pathogen; Coinf. > 2 refers to coinfections involving *M. genitalium* and at least two other pathogens.

	Positive to MG on Total Population	Total Coinfection	Coinf. 2	Coinf. > 2	MH	TV	CT	NG	UP	UU
Total	118 (7.4%)	57 (48%)	35 (30%)	22 (19%)	23 (19%)	0	11 (9%)	7 (6%)	11 (9%)	34 (29%)
	Positive to MG	Total coinf./pos on respective population								
Male	102 (10.2%)	44 (43%)	28 (27%)	16 (15%)	16 (15%)	0	9 (8%)	7 (6%)	4 (3%)	28 (24%)
Female	16 (2.6%)	13 (81%)	7 (43%)	6 (37%)	7 (43%)	0	2 (12%)	0	7 (43%)	6 (37%)

Abbreviations: MH, *Metamycoplasma hominis*; TV, *Trichomonas vaginalis*; CT, *Chlamydia trachomatis*; NG, *Neisseria gonorrhoeae*; UP, *Ureaplasma parvum*; UU, *Ureaplasma urealyticum*.

## Data Availability

Available upon reasonable request.

## Abbreviations

The following abbreviations are used in this manuscript:

MG	*Mycoplasma genitalium*
STIs	Sexually transmitted infections
NGU	Nongonococcal urethritis
PID	Pelvic inflammatory disease
MSM	Men who have sex with men
NAATs	Nucleic Acid Amplification Tests
VNTR	Variable number tandem repeats
IC	Internal Control
ML	Maximum likelihood
F	Forward
R	Reverse
MH	*Metamycoplasma hominis*
TV	*Trichomonas vaginalis*
CT	*Chlamydia trachomatis*
NG	*Neisseria gonorrhoeae*
UP	*Ureaplasma parvum*
UU	*Ureaplasma urealyticum*
MM	*Master mix*
AOUI	University Hospital
ECDC	European Centre for Disease Prevention and Control
AMR	Antimicrobial resistance
